# BCG vaccination in children with severe combined immunodeficiency in a tertiary center: evaluation of complications and risks

**DOI:** 10.1016/j.jped.2024.09.008

**Published:** 2024-12-22

**Authors:** Matheus Henrique Botaro, Jorgete Maria e Silva, Soraya Regina Abu Jamra, Stephanie Zago Geraldino, Persio Roxo-Junior

**Affiliations:** Universidade de São Paulo, Faculdade de Medicina de Ribeirão Preto, Departamento de Pediatria, Divisão de Imunologia e Alergia Pediátrica, Ribeirão Preto, SP, Brazil

**Keywords:** Severe combined immunodeficiency, Immunologic deficiency syndromes, BCG vaccine, Adverse reactions, Attenuated vaccines, Neonatal screening

## Abstract

**Objective:**

To describe the complications and risks associated with BCG (Bacillus Calmette-Guérin) vaccination in patients diagnosed with SCID (Severe Combined Immunodeficiency).

**Methods:**

This is a descriptive case series study. Medical charts were retrospectively reviewed for demographics, clinical manifestation, laboratory findings at diagnosis, outcome, and diagnosis of BCG vaccine-associated complications.

**Results:**

Eleven patients diagnosed with SCID were enrolled. Ten were male. Seven (64 %) were considered probable SCID, while four (36 %) were considered definite SCID (genetically confirmed). The median age at the onset of symptoms was one month; the median age at SCID diagnosis was four months. Respiratory symptoms were the most frequent. Eight patients were vaccinated within seven days of life. Seven (87 %) of these patients experienced BCG vaccine-associated complications (86 % disseminated reactions; 14 % localized reactions). BCG vaccine-associated complications were the first clinical manifestation in 75 % of the vaccinated patients. Less than half of the patients (36 %) underwent hematopoietic stem cell transplantation. The overall death rate was elevated (73 %); the death rate related to BCG vaccination was 25 %.

**Conclusions:**

Patients with SCID can present a high rate of BCG vaccine-associated complications, which negatively impact the clinical outcome and mortality. Pediatricians must be aware that BCG vaccine-associated complications can be the first presentation and a warning sign of SCID. Implementing newborn screening for SCID in Brazil may represent a worthy opportunity to impact the health outcomes of affected infants significantly.

## Introduction

Inborn Errors of Immunity (IEI) are a heterogeneous group of genetic diseases that result in immune dysregulation and immunodeficiency. The clinical manifestations of these diseases are heterogeneous and include greater susceptibility to infections, allergies, neoplasms, and autoimmunity. Until 2022, approximately 485 clinical phenotypes of IEI had been described.^1^

Severe Combined Immunodeficiency (SCID) is characterized by a profoundly impaired number or function of T lymphocytes, with CD3+ lymphocyte levels generally being lower than 300/mm^3^ of blood. SCID may also affect B cells, NK cells, or both. Although babies with SCID frequently seem healthy at birth, they are highly susceptible to severe infections, including opportunistic pathogens.^2^ SCID is considered rare, and its incidence is estimated at 1 case per 58,000 live births in the United States.^3^

SCID must be recognized early, and patients should be promptly referred to a specialized center for hematopoietic stem cell transplantation (HSCT). Pediatricians must be aware of the clinical and laboratory warning signs, like severe and persistent fungal infections in the neonatal period, infections by opportunistic pathogens, viral infections (by cytomegalovirus, Epstein–Barr virus, varicella, rotavirus, adenovirus, and respiratory syncytial virus) with severe and atypical evolution, chronic diarrhea, neuropsychomotor delay, failure to thrive, and family history of immunodeficiency. Some important laboratory signs include absolute peripheral lymphocyte count lower than 2500/mm³ in the neonatal period, absence of thymus in the chest X-ray, and severe and widespread reactions to vaccines with attenuated microorganisms, such as BCG (Bacillus Calmette-Guérin), rotavirus, oral poliomyelitis, measles, mumps, rubella, and chickenpox.^4^

The BCG vaccine is composed of an attenuated *Mycobacterium bovis* (Mb) strain and remains one of the most widely used vaccines worldwide. In most countries, it has routinely been administered as a protective strategy against meningitis and disseminated tuberculosis (TB) in children since the 1960s. In Brazil, the BCG vaccine is part of the nationwide vaccination schedule and is given to all children between birth and five years of age. The only contraindications are known/suspected immunosuppression, prematurity, and birth weight lower than 2000 g. The BCG vaccine may have localized (ulcer with a diameter greater than 1 cm, cold subcutaneous abscess, hot subcutaneous abscess, granuloma, non-suppurative regional lymphadenopathy greater than 3 cm, suppurative regional lymphadenopathy, keloid scar, and lupoid reaction) and disseminated (persistent fever, hepatosplenomegaly, pulmonary involvement, lack of weight gain, and appearance of lymphadenopathy in other lymph node chains) reactions.^5^

Because in most countries the BCG vaccine is administered at birth and many times before SCID is diagnosed, it is frequently associated with serious complications in SCID patients. A retrospective multicenter study conducted by Marciano et al. in 2014^6^ evaluated 349 SCID patients who were vaccinated with the BCG vaccine and demonstrated that 51 % of them had adverse reactions (34 % disseminated and 17 % localized reactions). Besides that, 46 deaths were associated with BCG vaccine complications.

Given that very few data for SCID and BCG vaccination have been published in Brazil, and that severe reactions to the BCG vaccine can warn about early SCID diagnosis, the authors aim to describe the complications and risks associated with BCG vaccination in SCID patients, to draw the attention of general pediatricians to this relevant warning sign.

## Methods

This is a descriptive case series study that evaluated SCID patients followed at the Division of Immunology and Allergy, Department of Pediatrics of Ribeirão Preto Medical School, University of São Paulo. For this study, all the data were collected between 2006 and 2021 and represent a 15-year cumulative experience.

SCID was defined according to the diagnosis criteria proposed by the European Society for Immunodeficiencies (ESID) and the Pan-American Group for Immunodeficiencies (PAGID).^7^ For the purposes of this retrospective study, the authors analyzed patients who received diagnoses of SCID on the basis of clinical and laboratory findings of recurrent/severe infections, failure to thrive, and severe T-cell lymphopenia.

Medical charts were retrospectively reviewed for the following data: clinical manifestation, age at the onset of symptoms, time elapsed between the onset of symptoms and referral, age at diagnosis, laboratory findings at diagnosis, age at HSCT, outcome, and diagnosis of BCG vaccine-associated complications. The latter complications were defined according to the classification of the Manual of Post-vaccination Adverse Events published by the Brazilian Ministry of Health. The complications were classified as localized (ulcer with a diameter larger than 1 cm, cold/hot subcutaneous abscess, regional suppurated lymphadenopathy, and lupus-like reaction) or disseminated (involvement of >1 organ) reactions.

The values of the means and medians were calculated with the Excel program.

This study was approved by the local Research Ethics Committee (Number: 4.718.864).

## Results

### Population demographics

Eleven SCID patients were included in the study. Ten were male. According to the year of diagnosis, 3/11 patients (27 %) were diagnosed before 2010, and 8/11 (73 %) were diagnosed after 2010. On the basis of the clinical, laboratory, and genetic data, 7/11 patients (64 %) were considered probable SCID (no genetic test), and 4/11 (36 %) were considered definite SCID (genetically confirmed).

Among the patients, 2/11 (18 %), who had not received the BCG vaccine, had suggestive or confirmed family history of SCID. One of these patients (patient IV) had a sibling diagnosed with SCID. The other patient (patient V) had two siblings (patients X and XI) who had died prematurely, aged four and five months, due to disseminated mycobacteriosis and presumptive diagnosis of SCID.

### Clinical manifestations

The median age at the onset of symptoms was one month (age range = from birth to seven months). The median time elapsed between the onset of symptoms and referral to the tertiary center was three months (time range = from zero to seven months). The median age at SCID diagnosis was five months (age range = from seven days to nine months). One patient had been diagnosed with SCID before the onset of symptoms because his family history suggested SCID.

The most frequent clinical manifestations were pneumonia, chronic cough, respiratory failure, and upper airway infections, verified in 5/11 patients (45 %), followed by failure to thrive in 2/11 patients (18 %), chronic diarrhea in 2/11 patients (18 %), delayed neuropsychomotor development in 1/11 patient (9 %), and neonatal sepsis in 1/11 patient (9 %).

### Laboratory tests and genetic analysis

At the time of diagnosis, 10/11 patients (90 %) had inadequate absolute lymphocyte counts for age-specific reference values.

Lymphocyte subsets and immunoglobulin levels were analyzed in 9/11 patients (82 %). All nine patients had CD3+ *T*-cell counts below the tenth percentile for age (P10, reference values for the Brazilian population). B-cell counts were below P10 in 6/9 patients (66 %) and normal in 3/9 (33 %). NK cell counts were below P10 in 4/9 patients (44 %) and adequate in 5/9 patients (55 %). T-cell functional assays were not performed.

Very low IgG, IgM, and IgA levels (below P3) were found in 3/9 patients (33 %). Isolated IgM level below P10 was found in 2/9 patients (22 %). Isolated IgG level below P10 was found in 1/9 patients (11 %). Combined IgM and IgA levels below P10 were found in 2/9 patients (22 %). Inadequate antibody response was found in 3/9 patients (33 %), all of whom presented very low IgG, IgM, and IgA levels.

Genetic test was performed in 4/11 patients (36 %) by new-generation sequencing. Nonsense variants were found in the genes *IL7RA, JAK3, IL2RG*, and *RAG1.* Segregation analysis was not accomplished.

Laboratory and genetic data are summarized in Table 1.

**Table 1:** Laboratory and genetic findings of the SCID patients.

Patient	Age (months)	Absolute number of lymphocytes /mm^3^	Lymphocyte subsets/mm^3^	Serum immunoglobulin levels (mg/dL)	Genotype
I	7	89	CD3^+^: 9 (< P10) CD19^+^: 74 (< P10) CD56^+^: 6 (< P10)	IgG: 7.56 (< P3) IgA: <6.28 (< P3) IgM: <5.36 (< P3)	NP
II	21	1080	CD3^+^: 483 (< P10) CD19^+^: 402 (< P10) CD56^+^: 195 (P10–50)	IgG: 704 (P25–50) IgA: 61.8 (P50–75) IgM: 58.5 (P3-P10)	NP
III	6	514	CD3^+^: 402 (< P10) CD19^+^: 79 (< P10) CD56^+^: 33 (< P10)	IgG: 402 (P10–25) IgA: 61.6 (> P97) IgM: 7.1 (< P3)	NP
IV	7 days	1600	CD3^+^: 13 (< P10) CD19^+^: 885 (P10–50) CD56^+^: 702 (P50–90)	IgG: 248 (< P3) IgA: 5.91 (< P3) IgM: 20.3 (< P3)	NP
V	6	1500	CD3+: 574 (< P10) CD19^+^: 66 (< P10) CD56^+^: 520 (P50–90)	IgG: 246 (< P3) IgA: 28.9 (> P97) IgM: 55 (> P97)	Pathogenic variant in *IL7-RA*
VI	3	3312	CD3^+^: 1908 (< P10) CD19^+^: 1116 (P10–50) CD56^+^: 288 (P10–50)	IgG: 435 (P25–50) IgA: 25.3 (P75–97) IgM: 83 (> P97)	NP
VII	10	805	CD3^+^: zero CD19^+^: 644 (< P10) CD56^+^: 161 (< P10)	IgG: 1160 (> P97) IgA: 6.6 (< P3) IgM: 29.1 (< P3)	Pathogenic variant in *JAK3*
VIII	7	647	CD3^+^: 13 (< P10) CD19^+^: 2 (< P10) CD56^+^: 632 (P50–90)	IgG: 23.3 (< P3) IgA: <6.4 (< P3) IgM: <4.5 (< P3)	Pathogenic variant in *RAG1*
IX	8	1063	CD3^+^: 64 (< P10) CD19^+^: 996 (P10–50) CD56^+^: 3 (< P10)	IgG: 386 (P10–25) IgA: <6.5 (< P3) IgM: 7.7 (< P3)	Pathogenic variant in *IL2RG*
X^a^	5	1000	NP	NP	NP
XI^a^	4	800	NP	NP	NP

a This pair of siblings had a presumptive SCID diagnosis because another sibling was later genetically diagnosed as SCID. They received the BCG vaccine and died prematurely due to disseminated mycobacteriosis, so the immunologic assessment could not be performed. NP: not performed.

### BCG vaccination and complications

The BCG vaccine was administered to 8/11 patients (73 %) within the first seven days of life, whereas 3/11 were not vaccinated (patient VI was not vaccinated due to prematurity and low weight at birth; patients IV and V were not vaccinated due to suggestive family history of SCID). All the vaccinated patients received the BCG Moreau/Rio de Janeiro strain as an intradermal injection in the right deltoid muscle. BCG vaccine-associated complications occurred in 7/8 vaccinated patients (87 %), who had not received early antimycobacterial therapy at the time of SCID diagnosis. The findings regarding BCG vaccination and its complications are shown in Table 2.

**Table 2:** BCG vaccination and associated complications in the SCID patients.

BCG complications among vaccinated patients (*n* = 8)	Yes 7 (87 %)	No 1 (13 %)
BCG complications according to genetic diagnosis (*n* = 7)	Probable SCID 4 (57 %)	Definite SCID 3 (43 %)
Type of complication (*n* = 7)	Local 1 (14 %)	Disseminated 6 (86 %)
Age at vaccination, days (*n* = 8)	First seven days 8 (100 %)	
Time elapsed between vaccination and complication, days (median, range) (*n* = 7)	53 days (7–384 days)	
BCG complication as first clinical manifestation (*n* = 8)	Yes 6 (75 %)	No 2 (25 %)

Among the patients that presented with disseminated complications, involvement of the lungs (wheezing, cough, dyspnea, and respiratory failure) was the main clinical presentation in all of them, followed by extra-regional lymphadenopathy, hepatosplenomegaly, and renal, cutaneous, and meningeal involvement. The only patient with local complication presented regional suppurated lymphadenopathy.

The Mb BCG strain was isolated in 5/7 patients (71 %), all of whom had disseminated complications. The sources of isolation were spread skin lesions, lungs, liver, spleen, lymph node, and stomach. In two patients (X and XI), Mb was isolated by necropsy.

### Supportive care

Among the patients enrolled in our study, 9/11 (82 %) used trimethoprim/sulfamethoxazole for *Pneumocystis jirovecii* prophylaxis and received intravenous immunoglobulin therapy before transplant. Besides that, these patients were advised to avoid sick contacts, live vaccines, and non-leukodepleted and irradiated blood products.

### Outcome

Among the patients, 8/11 (73 %) died. Five of them (62 %) had been vaccinated. At death, the patients were aged from four months to four years. The causes of death were identified in 7/8 patients (87 %): pulmonary sepsis (four patients), disseminated mycobacteriosis (two patients), and pneumonia due to chickenpox in one patient. Death related to the BCG vaccine was observed in 2/8 patients (25 %), both of whom died due to disseminated mycobacteriosis.

As for HSCT, 4/11 patients (36 %) underwent this procedure. The median age at HSCT was 11 months (age range = from 8.5 to 16 months). The median time elapsed between the onset of clinical manifestations and HSCT was nine months (time range = from 8.5 to 10 months). The median time elapsed between SCID diagnosis and HSCT was five months (time range = from three to eight months). One patient (25 %) was successfully transplanted but died three years later due to severe pneumonia by chickenpox. The three patients who did not die were successfully transplanted and are undergoing outpatient follow-up. One patient remains asymptomatic, and the other two patients present chronic cough due to pulmonary sequelae and are on oral and inhaled corticosteroids and antibiotic prophylaxis.

Among the seven patients who did not undergo HSCT, the overall mortality occurred at a median of nine months (age range = from 4 to 12 months). Regarding the causes of death, pulmonary sepsis occurred in 4/7 patients (57 %), and disseminated mycobacteriosis secondary to BCG occurred in 2/7 patients (28 %). The cause of death was not available for one patient.

## Discussion

This case series review focused on the complications related to the BCG vaccine to determine the clinical and epidemiological profile of SCID patients followed in a tertiary Pediatric Immunology center.

The prevalence of BCG vaccine-associated complications in the general population can vary widely. However, the prevalence of BCG vaccine-associated complications in SCID patients has been estimated to be higher than in the general population.^6^

Considering the SCID clinical complexity and severity, an important strategy to disseminate relevant knowledge about this disease is to characterize the initial clinical presentation, including complications related to BCG vaccination. Another important aspect is that a family history of severe complications in response to the BCG vaccine can offer an essential clue for early SCID diagnosis in infants.^8^ Prompt recognition of the warning signs of SCID will allow the patient to be referred to a tertiary reference center early and to be prescribed adequate treatment, contributing to a better prognosis.^9^ Furthermore, cell blood count and chest X-ray are easily available for general pediatricians to analyze and can be valuable for them to suspect SCID.

As in the case of our study, an active search for patients based on a standard collection form has been performed in other studies.^10^^,^^11^ Although SCID is considered a rare disease, the authors identified eleven patients with probable or definite diagnoses. Data obtained from the Latin American Society for Immunodeficiencies (LASID) Registry revealed that the estimated minimal incidence of SCID has been 0.12 cases per 100,000 after 1996.^12^ Therefore, the number of cases the authors obtained here may have been underestimated. The high complexity of the disease and physicians’ lack of knowledge about it can prevent prompt referral to a reference center. In our study, most of the patients were diagnosed after 2010, which likely reflected an overall improvement in disease recognition. In the last 20 years, the LASID Registry has been consolidated, and the Brazilian Group for Immunodeficiency has developed education programs for physicians, which has contributed to improving the recognition and diagnosis of SCID in Brazil. Nevertheless, much work remains to be done to increase awareness of SCID. Programs should target neonatologists, general pediatricians, intensive care specialists, and family physicians.

Although X-linked is considered the most common form of SCID, analysis of lymphocyte subsets showed that 6/9 patients (67 %) were T-B-, an immunophenotype that is more compatible with autosomal recessive inheritance. Two of these patients were diagnosed with *JAK3* and *RAG1* mutations*,* which are autosomal recessive genetic forms. A possible reason for this finding could be that four of our six T-B- patients were born from consanguineous marriages. Similarly, three cohort studies conducted in Brazil, Iran, and Greece demonstrated that autosomal forms predominated.^11^^,^^13^^,^^14^ The other 3/9 patients (33 %) were T-*B*+, suggesting an X-linked form. Two of these patients were diagnosed with *IL7RA* and *IL2RG* genetic forms.

Lymphopenia < 2500/mm³ at diagnosis was found in most of our patients (91 %). This laboratory finding has been described with a frequency of 90 % in other systematic reviews.^15^^,^^16^

The most frequent clinical manifestations were typical of SCID, as reported by other studies.^7^^,^^17^

Even though the median age at the onset of symptoms was one month, the median age at SCID diagnosis was five months. The median age at HSCT was high (around 11 months), which may have been due to difficulties in finding an adequate donor and a transplantation center early in Brazil. Moreover, no patients underwent transplantation before being aged 3.5 months, when the prognosis is thought to be better.^18^, ^19^, ^20^, ^21^ The authors found an elevated overall death rate (73 %) as well as a high rate of BCG vaccine-related death (25 %), also verified in other case series studies.^6^^,^^11^ Considering that SCID is a medical emergency, the long time elapsed between the onset of symptoms and diagnosis as well as the high age at transplantation may have contributed to the high death rate found in our study.

The prevalence of BCG-associated complications has been estimated to be higher in SCID patients than in the general population.^6^^,^^22^^,^^23^ Here, most of the vaccinated patients (87 %) experienced complications with the BCG vaccine (86 % disseminated and 14 % localized reactions). These patients did not receive early antimycobacterial therapy at the time of SCID diagnosis. It has been demonstrated that SCID patients who start antimycobacterial therapy while they are asymptomatic for BCG have significantly fewer BCG-associated complications.^6^ Lower rates of BCG vaccine-associated complications have been demonstrated by Marciano et al., 2014^6^ and Mazzuchelli et al., 2014,^11^ who found 51 % (34 % disseminated and 17 % localized reactions) and 65 % (74 % disseminated and 26 % localized reactions) of BCG vaccine-associated complications, respectively.

Another important finding of our study is that BCG complications were the first manifestation of SCID in 75 % of the vaccinated patients. These tables are higher than those presented in other case reports.^11^^,^^13^ This highlights that this warning sign is crucial for neonates, as shown by Roxo-Junior et al., 2013.^8^

Considering that many SCID patients are asymptomatic at birth and present high susceptibility to early death due to attenuated vaccine strains like BCG and that <20 % of these patients have a positive family history of SCID,^24^ some strategies have been proposed to reduce morbidity and mortality significantly, especially in countries that adopt BCG vaccination in the first weeks of life. Implementing newborn screening (NBS) for SCID represents a worthy opportunity to impact the health outcomes of affected infants substantially. This would provide early recognition and prompt interventions like avoiding BCG vaccination of suspected patients.^25^^,^^26^ However, NBS for SCID is routinely performed in fewer than 10 countries worldwide. In some European, Asian, and South American countries as well as Mexico, NBS is only carried out as a pilot project in regional centers. In Brazil, NBS for SCID has been routinely adopted in the city of São Paulo since 2020, in the Federal District since 2023, and in the state of Minas Gerais since February 2024. NBS represents a “new era” in SCID diagnosis, management, and prognosis.

Another strategy is to delay BCG vaccination. Marciano et al.^6^ proposed that until safer and more efficient forms of antituberculosis vaccines become available, delaying BCG vaccination beyond one month of age is likely to affect this highly vulnerable population favorably. Romanus et al.^22^ suggested that BCG vaccination should be postponed until six months of age in countries where the risk of neonatal TB is low. However, Brazil and other developing countries concentrate most of the TB cases worldwide, so the BCG vaccine is still considered important, especially against meningeal and miliary TB.^11^

The main limitations of the present study are the small number of patients (the study was conducted in a single center, and SCID is considered a rare disease) and the fact that genetic tests were conducted for a minority of the patients.

The survival and prognosis of SCID patients can be drastically compromised by late diagnosis and occurrence of serious infections early, including *M. bovis* dissemination after BCG vaccination.^6^^,^^8^^,^^11^ Therefore, in countries where the BCG vaccine is applied, pediatricians must be aware of its localized or disseminated complications and promptly refer patients for a specialized immunological workup.

In summary, our findings suggest that although SCID diagnosis has improved over the last decade, many patients are still referred and diagnosed late. Pediatricians must be aware that BCG vaccine-associated complications can be the first presentation of SCID and are highly frequent in SCID patients. These complications should be included as a warning sign of SCID diagnosis. Furthermore, patients with SCID presenting with BCG vaccine-associated complications are at increased risk of dying. Further multicenter studies focusing on the association between BCG vaccine-associated complications and SCID in the Brazilian population are needed.

## Funding source

This research did not receive any specific funding from public, commercial or non-profit sector funding agencies.

## Authors’ contributions

Matheus Henrique Botaro: Conception and design of the study, acquisition, analysis and interpretation of data, writing of the article, critical review of the relevant intellectual content, final approval of the version to be submitted.

Jorgete Maria e Silva: Conception and design of the study, acquisition, analysis and interpretation of data, writing of the article, critical review of the relevant intellectual content, final approval of the version to be submitted.

Soraya Regina Abu Jamra: Conception and design of the study, acquisition, analysis and interpretation of data, writing of the article, critical review of the relevant intellectual content, final approval of the version to be submitted.

Stephanie Zago Geraldino: Conception and design of the study, acquisition, analysis and interpretation of data, writing of the article, critical review of the relevant intellectual content, final approval of the version to be submitted.

Persio Roxo-Junior: Conception and design of the study, acquisition, analysis and interpretation of data, writing of the article, critical review of the relevant intellectual content, final approval of the version to be submitted.

## Conflicts of interest

The authors declare no conflicts of interest.
